# Diabetes and Hospitalizations Among Mexican Americans Aged 75 Years and Older

**DOI:** 10.1177/21501319241266108

**Published:** 2024-07-26

**Authors:** Garrett T. Coleman, Soham Al Snih

**Affiliations:** 1 The University of Texas Medical Branch, Galveston, TX, USA

**Keywords:** aging, hispanic, hospitalizations, type 2 diabetes mellitus, Mexican Americans

## Abstract

**Objective:**

To examine factors associated with hospitalization among Mexican Americans aged 75 years and older with diabetes (with and without complications) and without diabetes over 12 years of follow up.

**Methods:**

Participants (N = 1454) were from the Hispanic Established Population for the Epidemiologic Study of the Elderly (2004/2005-2016) residing in Arizona, California, Colorado, New Mexico, and Texas. Measures included socio-demographics, medical conditions, falls, depressive symptoms, cognitive function, disability, physician visits, and hospitalizations. Participants were categorized as no diabetes (N = 1028), diabetes without complications (N = 180), and diabetes with complications (N = 246).

**Results:**

Participants with diabetes and complications had greater odds ratio (1.56, 95% Confidence Interval = 1.23-1.98) over time of being admitted to the hospital in the prior year versus those without diabetes. Participants with diabetes had greater odds of hospitalization if they had heart failure, falls, amputation, and insulin treatment.

**Conclusions:**

In Mexican American older adults, diabetes and diabetes-related complications increased the risk of hospitalization.

## Introduction

In the United States, more than 1 in 6 adults are over the age of 65 years, with an average life expectancy of an additional 18.5 years. In 2020, 29.2% of adults over the age of 65 had diabetes^1^. From 2017 to 2020, the prevalence of diabetes among non-Hispanic whites was 13.6%, while the prevalence among Hispanics was 15.5%, with large variability among Hispanic origin subgroups.^1^ The increase in prevalence of diabetes among older adults can be attributed to an increase in life expectancy and to innovations in medications and management.^2^

Individuals with lower socioeconomic status and unemployment are at greater risk of developing Type 2 diabetes mellitus (T2DM), experience greater complications, and have lower life expectancy than those of higher socioeconomic status.^3^ As of 2018, Hispanics were 70% more likely to be diagnosed with diabetes and 1.3 times more likely to die from diabetes than non-Hispanic whites.^4^ The most significant risk factors associated with developing complications of diabetes include smoking, overweight/obesity, physical inactivity, elevated glycosylated hemoglobin, hypertension, and hypercholesterolemia.^1^ Hispanic adults with diabetes are more likely to have higher rates of albuminuria, worse glycemic control, and greater prevalence of metabolic syndrome compared to non-Hispanic whites. Additionally, compared to non-Hispanic whites, Hispanic adults are more likely to have diabetic retinopathy, nephropathy, lower extremity amputation, and are twice as likely to be hospitalized for end-stage renal disease.^4,5^ While relatively few studies have analyzed the factors associated with hospitalization in Hispanic American older adults with diabetes, several studies have analyzed the risk factors associated with hospitalization among the general population. In adults with T2DM, older age, polypharmacy, and diagnoses of hypertension, heart failure, myocardial infarction, sepsis, renal disease, and elevated HbA1C were associated with a higher likelihood of hospitalization within a year.^6-8^

Even though the prevalence of diabetes complications has generally trended down in the last 20 years, minorities continue to retain a disproportionate burden for complications related to diabetes.^5^ Moreover, several novel medications (e.g., glucagon-like peptide-1 receptor agonists, dipeptidyl peptidase 4 inhibitors, and sodium-glucose transport protein inhibitors) have demonstrated greater efficacy (especially for T2DM) and cardiovascular benefits than traditional anti-diabetes medications. However, Hispanics are more likely to have complications secondary to diabetes^3^ and are less likely to utilize novel medications to treat diabetes,^9^ particularly due to lack of access. Despite the increased prevalence of diabetes among Hispanic older adults, few studies have reported on the factors associated with hospitalization for this population. Using the Hispanic Established Population for the Epidemiologic Study of the Elderly (HEPESE), the objective of this study was to determine the factors associated with hospitalization among Mexican American older adults with and without diabetes over 12 years of follow up.

## Methods

### Procedures and Sample

Data were obtained from the HEPESE, which is comprised of a longitudinal cohort of Mexican Americans ≥65 years from the southwestern US (Arizona, California, Colorado, New Mexico, and Texas). HEPESE began in 1993/1994 (N = 3050) with in-home interviews every 2 or 3 years conducted by trained bilingual interviewers in Spanish or English, depending on the respondent’s preference. In 2004/2005, a new sample of respondents aged 75 years and older was added to the cohort of surviving participants (N = 1167) using sampling procedures similar to those for the original cohort. Information and data for the HEPESE are available at the National Archives of Computerized Data on Aging.^10^ For the present study, we used data collected from Wave 5 (hereafter referred as baseline) to Wave 9 (2004/2005-2016). Of 2069 eligible participants, we excluded 615 with incomplete information on diabetes, hospitalization, or any covariate at baseline, leaving a final sample size of 1454 (Supplemental Figure 1). At the end of follow up, 263 participants were reinterviewed in person, 105 via proxy, 141 were lost to follow up/or refused to be reinterviewed, and 945 were confirmed deceased through the National Death Index or report from relatives. Compared to those included, excluded participants were significantly more likely to be older, unmarried, foreign-born; to have a lower level of education; to report diabetes and diabetes complications (kidney, eye, circulation, and amputation), stroke, hip fracture, high depressive symptoms, limitations in activities and daily living; to be hospitalized in the previous year; and to have lower scores on the Mini Mental Status Examination (MMSE). The university’s institutional review board approved the study protocol, and oral informed consent was obtained from each participant at the time of the interview.

## Measures

### Primary Predictor Variable

Diabetes status was assessed by asking participants “have you ever been told by a doctor that you have diabetes, sugar in your urine, or high blood sugar?” (Yes vs No). Participants who reported a diabetes diagnosis were asked about disease duration (<10 years vs ≥10 years), if they followed a diet for diabetes (Yes vs No), and treatment received (unknown, oral hypoglycemic, insulin, or oral hypoglycemic/insulin combination). Participants were asked if, as a result of their diabetes, they have ever had any problems with their kidneys or eyes (micro-complications), or circulation or any amputations (macro-complications; Yes vs No).

### Covariates

Sociodemographic variables included in the model were age, sex, marital status (married vs unmarried), years of formal education, language of interview (English or Spanish), nativity (US-born vs foreign-born), and financial strain (great deal/some vs little/none).

Health measures and behaviors included in the model were self-reported medical conditions (hypertension, arthritis, heart failure, heart attack, stroke, cancer, or hip fracture), body mass index (BMI) was calculated as measured weight in kilograms divided by measured height in squared meters, depressive symptoms measured with the Center for Epidemiologic Studies Depression Scale (CES-D ≥ 16)^11,12^; cognitive function assessed with the MMSE^13^; fall status (none vs ≥ 1 falls) in the last year; disability as ≥1 limitations in activities of daily living (ADLs—walking across a small room, bathing, grooming, dressing, eating, transferring from a bed to a chair, and using the toilet)^14^; and physician visits (0 vs ≥1) in the last year.

### Outcome

Hospitalizations were scored by asking “did you experience an illness or injury (get sick or get hurt) that required staying overnight or longer in a hospital (not a nursing home)?” (Yes vs No).

### Statistical Analyses

Participants were divided in 3 groups: no diabetes (N = 1028), diabetes without complications (N = 180), and diabetes with complications (N = 246). Chi-square, Fischer’s Exact, and ANOVA tests were used to compare the sample characteristics by diabetes status at baseline. We used the Generalized Estimating Equation (GEE) models using the GENMOD procedure in SAS software to estimate the odds ratio (OR) and 95% confidence interval (CI) of hospitalizations over 12-years of follow-up as a function of diabetes group, controlling for all covariates. The models used a logit link binomial distribution and unstructured correlation structure to account for repeated measures of participants. The covariance matrices for GEE were chosen based on the Akaike information criterion and Bayesian information criterion values. We used GEE models because of their several advantages: (a) the number of repeated measurements per study participant is not required to be constant over time; (b) the assessment of times does not need to be the same across study participants; (c) the GEE approach can be applicable to several types of response variables (binary, ordinal, discrete, and counts); (d) continuous and categorical variables can be used as time-dependent and time-independent variables; and (e) the GEE uses an all available pairs method to handle missing data.^15^ All variables, including diabetes status /complications, were analyzed as time-varying (potential to change from interview to interview), except for sex, education, and nativity. Participants who died, refused to participate, or were lost to follow up were included until their last follow-up date (last interview date over the 12-year follow up). Additionally, we analyzed the subsample who reported diabetes at baseline with and without complications (N = 426) to examine the factors associated with hospitalizations over time. All analyses were performed using SAS version 9.4 (SAS Institute, Inc., Cary, NC, USA).

## Results

Table 1 presents the descriptive characteristics of the overall sample and by diabetes status/complications. The overall mean age of the sample was 81.3 ± [standard deviation (SD) 4.7] years, most of the sample were female; married; born in the US; and had a lower level of education. The most common medical conditions were hypertension, arthritis, and diabetes. The mean BMI and MMSE scores were 24.5 ± 4.9 kg^2^ and 22.4 ± 5.8, respectively. Compared to those without diabetes, those with diabetes and complications were younger; more likely to report hypertension, stroke, heart attack, heart failure, ADL disability, depressive symptoms, or falls; had a higher BMI; and more likely in the last year to be hospitalized or have at least 1 medical doctor visit.

**Table 1.** Descriptive Characteristics of the Overall Sample and by Diabetes Subgroup/Complications (N = 1454).

Variables	Total	No diabetes	Diabetes with no complications	Diabetes with ≥ 1 complications	*P*-value
		N (%)	N (%)	N (%)	
Total	1454	1028	180	246	–
Mean age	81.3 ± 4.7	81.8 ± 5.0	80.0 ± 3.9	80.4 ± 3.8	<.0001
Female sex	894 (60.8)	626 (60.1)	113 (62.8)	155 (63.0)	.7714
Education	5.6 ± 3.9	5.6 ± 3.9	6.0 ± 3.9	5.3 ± 3.8	.2132
Married	655 (45.1)	449 (43.7)	90 (50.0)	116 (47.2)	.2226
Foreign-born	604 (41.5)	437 (42.5)	66 (36.7)	101 (41.1)	.3359
Spanish interview	1170 80.5)	820 (79.8)	142 (78.9)	208 (84.6)	.2000
Financial strain	846 (58.2)	587 (57.1)	102 (56.7)	157 (63.8)	.1438
Mean BMI,	27.5 ± 4.9	27.0 ± 4.7	28.5 ± 4.5	29.0 ± 5.3	<.0001
Hypertension	894 (61.5)	571 (55.5)	126 (70.0)	197 (80.1)	<.0001
Arthritis	863 (59.4)	599 (58.3)	102 (56.7)	162 (65.9)	.0690
Heart failure	380 (26.1)	234 (22.8)	48 (26.7)	98 (39.8)	<.0001
Heart attack	123 (8.5)	70 (6.8)	16 (8.9)	37 (15.0)	.0002
Stroke	105 (7.2)	63 (6.1)	9 (5.0)	33 (13.4)	.0002
Cancer	101 (7.0)	64 (6.2)	17 (9.4)	20 (8.1)	.2126
Hip fracture	53 (3.7)	41 (4.0)	4 (2.2)	8 (3.3)	.5571
CES-D ≥ 16	243 (16.7)	162 (15.8)	21 (11.7)	60 (24.4)	.0008
ADL disability	449 (30.9)	288 (28.0)	50 (27.8)	111 (45.1)	<.0001
MMSE score	22.4 ± 5.8	22.3 ± 5.9	23.1 ± 5.3	21.9 ± 5.9	.0860
Falls	457 (31.4)	300 (29.2)	54 (30.0)	103 (41.9)	.0005
Hospitalization	353 (24.3)	221 (21.5)	40 (22.2)	92 (37.4)	<.0001
Physician visits	1313 (90.3)	906 (88.1)	172 (95.6)	235 (95.5)	<.0001
Deceased	946 (65.1)	639 (62.2)	121 (67.2)	186 (75.6)	.0003

Abbreviations: ADL, activities of daily living; BMI, body mass index; CES-D, Center for Epidemiologic Studies Depression Scale; MMSE, mini mental status examination.

Supplemental Table 1 presents the descriptive characteristics of the participants with diabetes (N = 426). Approximately 64% had disease duration for more than 10 years, 72.5% followed a diet for diabetes, 90.4% reported taking anti-diabetic oral medications, 9.6% reported using insulin, and 14.8% reported using both oral medications and insulin. In all, 57.8% had at least 1 complication secondary to diabetes, including circulatory complications, eye complications, renal complications, and an amputation. Participants with diabetes and complications had the greatest hospitalization rate. However, this rate fell from 37.4 % to 27.9 %. The percent of hospitalizations among those with diabetes and without complications ranged from 22.2% to 35.6% (Supplemental Figure 2).

Table 2 presents the GEE model for hospitalization over time as a function of diabetes status/complication in the overall sample. The odds of hospitalization among participants with diabetes complications was 1.56 times greater than the odds of participants without diabetes, after controlling for all covariates. Other factors associated with hospitalization over time included heart failure, heart attack, stroke, cancer, hip fracture, ADL disability, falls, and have at least 1 medical doctor visit.

**Table 2.** Generalized Estimating Equation Model for Hospitalization Over Time as a Function of Diabetes Status/Complication in the Overall Sample (N = 1454).

Predictor variables	OR (95% CI)	*P*-value
Time	1.00 (0.96-1.03)	.7777
No diabetes	Reference	
Diabetes without complications	1.08 (0.84-1.39)	.5480
Diabetes with complications	1.56 (1.23-1.98)	.0003
Age	1.03 (0.98-1.03)	.8191
Female	1.16 (0.94-1.45)	.1688
Education	0.98 (0.96-1.01)	.2510
Married	1.18 (0.96-1.44)	.1103
Foreign-born	1.11 (0.91-1.34)	.3039
Spanish interview	1.29 (0.98-1.68)	.0651
Financial strain	1.11 (0.92-1.34)	.2814
BMI (Kg/m^2^)	0.99 (0.98-1.01)	.4856
Hypertension	1.07 (0.88-1.31)	.4882
Arthritis	0.94 (0.78-1.14)	.5456
Heart failure	1.87 (1.52-2.30)	<.0001
Heart attack	1.71 (1.23-2.39)	.0015
Stroke	2.20 (1.54-3.15)	<.0001
Cancer	1.56 (1.15-2.10)	.0041
Hip fracture	1.83 (1.17-2.85)	.0076
Depressive symptoms (CES-D ≥ 16)	1.23 (0.98-1.55)	.0704
ADLs	1.29 (1.06-1.57)	.0100
MMSE	0.99 (0.98-1.01)	.6203
Falls	1.64 (1.37-1.97)	<.0001
Physician visits	3.43 (1.98-5.98)	<.0001

Abbreviations: ADL, activities of daily living; BMI, body mass index; CES-D, Center for Epidemiologic Studies Depression Scale; CI, confidence Interval; MMSE, Mini Mental Status Examination; OR, odds ratio.

Table 3 presents the GEE model for hospitalizations over time in those with diabetes (N = 426). The factors associated with hospitalization included being married, heart failure, falls, amputation, and insulin treatment.

**Table 3.** Generalized Estimating Equation Model for Hospitalization Over Time in Those With Diabetes (N = 426).

Predictor variables	OR (95% CI)	*P*-value
Time	1.01 (0.88-1.16)	.8726
Age	1.01 (0.91-1.11)	.9014
Female	1.46 (0.78-2.76)	.2386
Education	0.97 (0.91-1.04)	.4357
Marriage	1.86 (1.07-3.25)	.0279
Foreign-born	1.08 (0.61-1.91)	.7925
Spanish interview	1.38 (0.59-3.24)	.4564
Financial strain	1.04 (0.62-1.76)	.8762
BMI	1.01 (0.98-1.04)	.6523
Hypertension	1.11 (0.55-2.25)	.7705
Arthritis	1.22 (0.75-2.01)	.4238
Heart failure	2.15 (1.27-3.63)	.0042
Heart attack	1.07 (0.47-2.44)	.8681
Stroke	1.46 (0.57-3.78)	.4295
Cancer	1.73 (0.74-4.07)	.2057
Hip fracture	0.84 (0.17-4.04)	.8267
Depressive symptoms (CES-D ≥ 16)	1.41 (0.85-2.56)	.1710
MMSE	0.99 (0.94-1.04)	.6057
ADLs	1.06 (0.61-1.83)	.8413
Falls	1.84 (1.13-3.00)	.0147
Physician visits	0.87 (0.23-3.33)	.8414
Disease duration (≥10 years)	0.78 (0.46-1.33)	.3620
Kidneys	1.75 (0.87-3.52)	.1195
Eyes	0.79 (0.41-1.28)	.3396
Circulation	1.10 (0.64-1.89)	.7436
Amputation	3.38 (0.85-13.44)	.0836
Insulin	2.34 (1.20-4.55)	.0123
Oral hypoglycemics	0.99 (0.44-2.23)	.9840

Abbreviations: ADL, activities of daily living; BMI, body mass index; CES-D, Center for Epidemiologic Studies Depression Scale; CI, confidence Interval; MMSE, Mini Mental Status Examination; OR, odds ratio.

## Discussion

Our study examined the factors associated with hospitalization among Mexican Americans aged 75 years and older without diabetes and with diabetes (with and without complications) over 12 years of follow up. The percent of hospitalizations ranged from 24.3% to 25.9% throughout the follow up. Participants with diabetes and complications had 61.1% greater odds over time of being admitted to the hospital in the previous year compared to those without diabetes, after controlling for all covariates. Factors such as heart failure, heart attack, stroke, cancer, hip fracture, ADL disability, falls, and physician visits were associated with the likelihood of hospitalization over time. In participants with diabetes, heart failure, falls, amputation, and receipt of insulin treatment increased the odds of hospitalization over time.

Overall, our findings were consistent with current research with 2 notable exceptions.^6-8^ In our study, participants with diabetes who reported a fall in the last year had 64.8 % greater odds of being admitted to the hospital in the prior year, and participants with an amputation had 77.2 % greater odds of being admitted to the hospital in the prior year.

Several mechanisms may explain our findings. For example, the pathophysiology between having diabetes and increased falls is unclear; however, several risk factors have been identified. Older adults are at increased risk of falls due to impairments in vision, visual perception, balance, gait, mobility, and muscle strength.^16^ Several complications of diabetes exacerbate these risk factors for falls. For example, diabetic retinopathy impairs vision and visual perception. Diabetic neuropathy impairs gait and mobility and is associated with reduced reaction time.^17^ Individuals with diabetes experience decreased muscle mass secondary to increased insulin resistance, hyperglycemia, myosteatosis, and peripheral neuropathy.^18,19^ Insulin resistance and poor glycemic control can result in postural hypotension and/or dizziness, resulting in decreased balance.^20^

The pathophysiology for amputation secondary to diabetes is well documented. Approximately 84% of diabetes-related lower extremity amputations are a result of diabetic foot ulcers. Patients with diabetes have greater risk of foot ulceration due to peripheral artery disease and diabetic neuropathy; and experience poor sensation due to diabetic neuropathy. In 2022, there were over 154 000 diabetes-related amputations in the US. An individual who has had an amputation has a lower likelihood of 5-year survival than someone with coronary artery disease, breast cancer, or colorectal cancer.^21^ Minority groups experience a greater risk of lower extremity amputation.^22^ Hispanics are 2.17 times more likely to have a diabetic foot amputation than non-Hispanic whites.^23^ Previous lower extremity amputation, level of lower extremity amputation, and patient comorbidities are significant risk factors contributing to re-ulceration, re-amputation, mortality, and declining functional status in those with diabetes.^23^ Amputation is both a complication of diabetes and an indicator of disease progression.^24^ Individuals with a prior amputation are more likely to have poor glycemic control, peripheral neuropathy, and peripheral arterial disease.^25^ Peripheral arterial disease has been found to be a marker for systemic vascular disease that involves coronary, cerebral, and renal vessels, all factors leading to an elevated risk of myocardial infarction, stroke, and death.^26^

Our study had some limitations. First, the survey did not include information related to participants’ glycemic control and thus we were unable to definitively determine whether participants’ diabetes was controlled. Second, our data was limited in determining participants’ treatment regimen for diabetes (ie, anti-diabetic oral medications vs injectable insulin) and did not include participants’ specific medications. Third, hospitalizations were obtained by self-report only and were not verified through medical records. Fourth, our exclusion group at baseline could have resulted in underestimating the relationship with factors associated with hospitalization. Fifth, our findings from individuals 75 years and older could be affected by recall bias. Finally, our findings are only generalizable to all the Mexican Americans living in the Southwest and not to the larger Hispanic population in the US, which is heterogeneous in prevalence of diabetes.^27^ The strengths of our study included a focus on the understudied population of Mexican American older adults aged 75 years and older, who have higher rates of diabetes than the national average. Additionally, our sample had a long follow up of 12 years. Our analysis was novel in the use of analyzing complications as time varying.

## Conclusions

Mexican American older adults with diabetes and diabetes-related complications are at higher risk of hospitalization. Heart failure, stroke, insulin use, falls, and amputation were associated with greater odds of hospitalization over time. Many of these complications are preventable with proper screening of diabetes-related complications. Removing barriers and improving access to primary care and appropriate screening can help reduce the risk of diabetes-related complications and comorbidities, including the incidence of hospitalization.^28^

## Supplemental Material

sj-docx-1-jpc-10.1177_21501319241266108 – Supplemental material for Diabetes and Hospitalizations Among Mexican Americans Aged 75 Years and Older Supplemental material, sj-docx-1-jpc-10.1177_21501319241266108 for Diabetes and Hospitalizations Among Mexican Americans Aged 75 Years and Older by Garrett T. Coleman and Soham Al Snih in Journal of Primary Care & Community Health
