# Supplementary material for: Diabetes and Hospitalizations Among Mexican Americans Aged 75 Years and Older
Source: J Prim Care Community Health. 2024 Jul 26;15:21501319241266108. doi: 10.1177/21501319241266108 (PMC11282514; doi:10.1177/21501319241266108)
Supplement: sj-docx-1-jpc-10.1177_21501319241266108 – Supplemental material for Diabetes and Hospitalizations Among Mexican Americans Aged 75 Years and Older [file sj-docx-1-jpc-10.1177_21501319241266108.docx]

**Supplemental Figure 1. Flow chart of participant selection.**

Baseline Sample

(2004/05)

N=2069

Diabetes=8

Missing information on any covariates at baseline=607

Final Sample

(2004/05)

N=1454

**Wave 6 (2007/08)**

Interviewed, n=1084

Deaths, n=205

Refused/lost to follow-up, n=165

**Wave 7 (2010/11)**

Interviewed, n=784

Deaths, n=297

Refused/lost to follow-up, n=168

**Wave 8 (2012/13)**

Interviewed, n=558

Deaths, n=189

Refused/lost to follow-up, n=205

**Wave 9 (2016)**

Interviewed, n=368

Deaths, n=254

Refused/lost to follow-up, n=141

**Supplemental Table 1: Descriptive Characteristics of Participants with Diabetes (N=426).**

| Variables | N (%) |
| --- | --- |
| Total | 426 |
| Duration |  |
| < 10 Years | 154 (36.2) |
| ≥ 10 Years | 272 (63.9) |
| Diet |  |
| No | 117 (27.5) |
| Yes | 309 (72.5) |
| Insulin |  |
| No | 322 (75.6) |
| Yes | 104 (24.4) |
| Pills |  |
| No | 41 (9.6) |
| Yes | 385 (90.4) |
| Treatment |  |
| Pills | 322 (75.6) |
| Insulin | 41 (9.6) |
| Insulin + Pills | 63 (14.8) |
| Kidney |  |
| No | 370 (86.9) |
| Yes | 56 (13.2) |
| Eyes |  |
| No | 273 (64.1) |
| Yes | 153 (35.9) |
| Circulation |  |
| No | 247 (58.0) |
| Yes | 179 (42.0) |
| Amputation |  |
| No | 407 (95.5) |
| Yes | 19 (4.5) |
| Any Complications |  |
| No | 180 (42.3) |
| Yes | 246 (57.8) |

**Supplemental Figure 2: Percent of hospitalizations among subgroups over time 9 (N=1454).**

Note: Interview dates for each Wave are: Wave 5 = 2004/05; Wave 6 = 2007/08; Wave 7 = 2010/11; Wave 8 = 2012/13; Wave 9 = 2016.
